# Biomechanical Changes and the Time Course of Recovery in Lower Extremities of Recreational Runners Following a Simulated Treadmill Half-Marathon

**DOI:** 10.1186/s40798-025-00824-x

**Published:** 2025-03-01

**Authors:** Wenjin Wang, Shulin Xu, Igor Komnik, Josef Viellehner, Marvin Zedler, Wolfgang Potthast

**Affiliations:** Institute of Biomechanics and Orthopedics, German Sport University Cologne, 50933 Cologne, Germany

## Abstract

**Background:**

Providing runners with injury prevention suggestions from a biomechanical perspective is crucial in light of the increased incidence of running-related injuries forecasted with the rising popularity of the half-marathon. Previous research has demonstrated associations between running injuries and patterns of lower extremity biomechanics, as well as indicating that inadequate rest between training sessions can result in injuries and underperformance. However, whether half-marathon running elicits lower extremity biomechanical changes and the recovery time remains largely unclear. This study aimed to investigate the acute changes (pre-run and immediately post-run) and the time course of recovery (pre-run, day 1, and day 2) in neuromuscular function, landing strategies, and lower extremity joint mechanics of recreational runners following a simulated half-marathon protocol on a treadmill.

**Results:**

Compared to pre-half-marathon measurements, we observed significant reductions in concentric (Flexors: Pre: 1.49 ± 0.50Nm/kg, Post: 1.34 ± 0.54Nm/kg, *p* < 0.001; Extensors: Pre: 2.19 ± 0.73Nm/kg, Post: 2.00 ± 0.82Nm/kg, *p* < 0.001) and eccentric (Flexors: Pre: 1.67 ± 0.68Nm/kg, Post: 1.34 ± 0.62Nm/kg, *p* < 0.001; Extensors: Pre: 2.44 ± 0.13Nm/kg, Post: 1.96 ± 0.12Nm/kg, *p* < 0.001) torques of the knee flexors and extensors, reductions in eccentric knee flexor to concentric knee extensor torque ratios (Pre: 0.78 ± 0.27, Post: 0.68 ± 0.22, *p* < 0.001), and impaired knee (Pre: 1.6 ± 0.1°, Post: 3.0 ± 0.2°, *p* < 0.001) and hip (Pre: 1.5 ± 0.2°, Post: 2.6 ± 0.2°, *p* < 0.001) joint position sense immediately post running. Additionally, we observed an increase in contact time (*p* = 0.006), decreases in peak vertical ground reaction force (*p* < 0.001) and impulse (*p* < 0.001), and changes in lower extremity joint kinematics and kinetics during the stance phase of running immediately after the half-marathon. Most measured parameters recovered within one day, except hip joint position sense, which was restored within two days. By the second day, we also observed super-compensation in thigh muscle torques.

**Conclusion:**

The study revealed that simulated treadmill half-marathon induces alterations to neuromuscular function, impacts landing strategies, and elicits changes in lower extremity joint mechanics. However, these effects are temporary and resolve within two days post-run. These findings provide valuable insights to optimize training responses and prevent overtraining in recreational runners.

## Key Points

Half-marathon running induces alterations in neuromuscular function, impacts landing strategies, and elicits changes in lower extremity joint mechanics.

Most measured parameters returned to pre-marathon status within one day, except for hip joint position sense, which was restored within two days. By the second day, we observed super-compensation in thigh muscle torques.

We recommend that runners avoid engaging in higher-intensity physical activities within two days after completing the half marathon, as they are at a greater risk of injury during this time window.

## Background

The Berlin half-marathon participants increased more than sevenfold from 1990 to 2023; this surge in popularity has been accompanied by a rise in running-related musculoskeletal injuries [1], with a prevalence rate of 44.6% ± 18.4% [2]. The majority of running-related injuries are classified as overuse injuries [3, 4], with runners accumulating over 12,600 repetitive foot strikes [5] during a half-marathon. This repetitive activity leads to fatigue, which reduces neuromuscular control, and compromises mechanical stability, thereby exacerbating the risk of musculoskeletal injuries [6]. Research has shown a direct relationship between biomechanical risk factors and running-related injuries, with significant differences in lower extremity kinematics, kinetics and proprioception observed among affected runners [4, 7–9]. Previous research has highlighted impairments in muscle torque and joint kinematics following long-distance running [7, 10, 11]. Furthermore, recovery after endurance running is a multifaceted process [12]. A previous study indicated that the maximal voluntary contraction of the knee extensor muscles recovered two days after completing a marathon [13]. However, there is limited understanding of the recovery time for biomechanical variables after a half-marathon. Inadequate rest between training sessions can result in injuries and underperformance [14]. Recreational runners constitute the majority of half-marathon participants and face unique challenges [15], including a high risk of overuse injuries due to limited access to professional guidance. Therefore, this study aimed to investigate the acute changes (pre-run and immediately post-run) and the time course of recovery (pre-run, day 1, and day 2) in neuromuscular function, landing strategies, and lower extremity joint mechanics of recreational runners following a simulated half-marathon protocol conducted on a treadmill. Understanding the changes and recovery time for these parameters can optimize training responses and prevent overtraining for recreational runners from a biomechanical perspective.

## Methods

### Participants

We recruited 36 recreational runners (mean age: 28.5 ± 5.6 years; body mass: 64.0 ± 10.5 kg; height: 171.7 ± 8.0 cm), comprising 19 females (mean age: 26.2 ± 4.3 years; body mass: 58.3 ± 8.5 kg; height: 167.2 ± 6.9 cm) and 17 males (mean age: 31.1 ± 5.8 years; body mass: 70.4 ± 8.7 kg; height: 176.9 ± 5.9 cm). The sample size was calculated using G*Power software. An a priori calculation was conducted for repeated-measures ANOVA with an effect size of 0.25, a significance level of 0.05, and a power of 0.80. For the acute change analysis (2 time points: pre-run and immediately post-run) and the recovery time analysis (3 time points: pre-run, day 1, and day 2), a minimum of 28 participants was required. Recreational runners were defined as individuals with more than six months of regular running training (i.e. at least one day per week), running 10–50 km per week primarily for health or leisure purposes [16]. Inclusion criteria were as follows: age 20–40 years; completion of at least one half-marathon in the past two years; engagement in running one to three times per week for at least six months; a minimum of 20 km per week of distance running; active running primarily for health or leisure purposes; rear-foot striking gait; and no significant injuries in the past six months. Participants were recruited through online advertisements posted in local running clubs and flyers distributed at the German Sport University Cologne. The study protocol was reviewed and approved by the Institutional Review Board of the German Sport University Cologne (No. 141/2022), and written informed consent was obtained from each participant prior to the investigation.

### Experiment Protocol

To explore the acute changes in neuromuscular function, landing strategies, and lower extremity joint mechanics following a simulated half-marathon protocol on a treadmill, we measured above parameters pre and immediately post a simulated treadmill half-marathon. To investigate the time course of recovery, these parameters were also assessed one day and two days post running.

#### Muscular Strength Assessment 

**Fig. 1:**
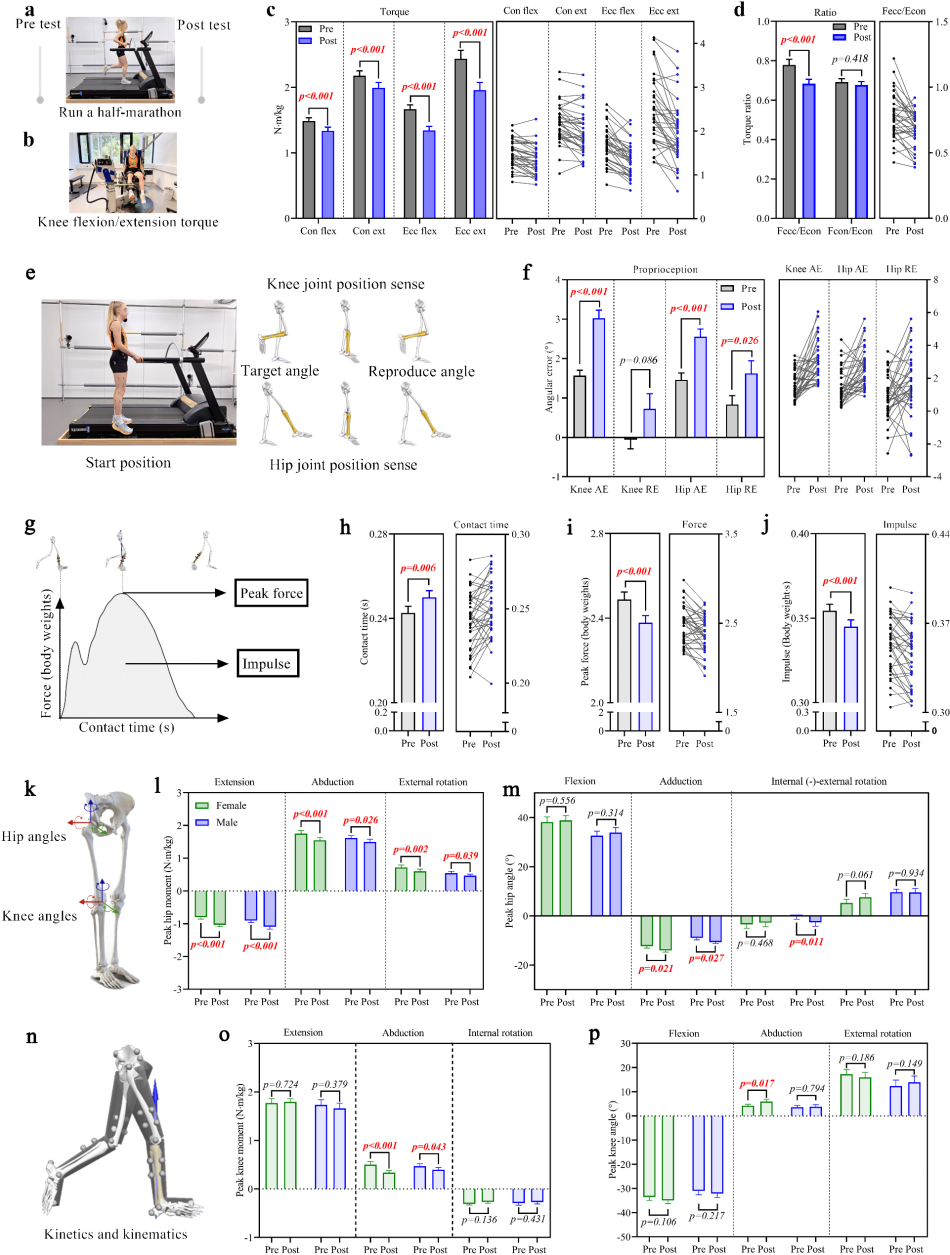
Comparative analysis of biomechanical variables pre and post half-marathon running. **a**, Thirty-six recreational runners (19 females and 17 males) completed a simulated half-marathon running on an instrumented treadmill at their preferred speed. **b**, Concentric and eccentric knee flexor and extensor torques were measured on the right leg, with five repetitions, at an angular velocity of 60°/s using an Isomed 2000 isokinetic dynamometer. **c**, Peak torque for concentric and eccentric movements of the knee flexor and extensor. **d**, Functional eccentric knee flexor to concentric knee extensor peak torque ratios (Fecc/Econ), and concentric knee flexor to concentric knee extensor peak torque ratios (Fcon/Econ). The peak torque of the middle three repetitions was used for knee flexors and extensors for statistical analysis (**c**,** d**). **e**, Knee and hip joint position sense was assessed. **f**, Joint position sense for hip/knee flexion was evaluated using the active reproduction technique, with absolute (AE) and relative (RE) angular errors calculated between the target and replicated angles. The mean value of three trials was used for AE and RE calculations (**f**). **g**, An example of contact time, peak force and impulse. **h**, Contact time, **i**, peak vertical ground reaction forces, and **j**, impulse during the stance phase of running. **k**, Local definitions of joint motions of the hip and knee joints. **l**, Peak hip moments and **m**, peak hip angles during the stance phase of running. **n**, Measurements were taken for knee/hip angles and moments during a separate running task (10 km/h) pre and post-half-marathon. **o**, Peak knee moments and **p**, peak knee angles during the stance phase of running. The peak average value of ten consecutive gait cycles was used for analysis (**h**,** i**,** j**,** l**,** m**,** o**,** p**). Data are presented as mean ± SEM (Standard error of the mean). The error bars represent the SEM of the data across participants. Statistical analyses were conducted using two-way repeated measures ANOVA

After an explanation of the experimental protocol, participants completed a five-minute self-directed warm-up. Concentric and eccentric knee flexor and extensor torques were measured at an angular velocity of 60°/s using an Isomed 2000 isokinetic dynamometer (D&R GmbH, Hemau, Germany). Participants were seated with back and leg support (Fig. 1b). The dynamometer’s position and height were adjusted to align the knee flexion axis with the arm axis of the dynamometer. Straps across the thorax, waist, and thigh secured the participants for stabilization, allowing the knee a range of motion from 10° to 90°. The testing was conducted on the right leg, with five repetitions. Verbal encouragement and visual feedback of knee torque were provided during all tests.

#### Proprioception Assessment 

Reflective markers were positioned over the bony landmarks (Fig. 1e). All markers were secured with skin-medical tape to prevent them from falling off during the run. The lower-body marker-set comprising seven anatomic segments was tracked, including the pelvis, bilateral thighs, shanks, and feet (Fig. 1h). Data collection with a static calibration trial. The joint position sense of the hip and knee in the right leg was assessed using the active reproduction technique, following a previously published protocol [17]. Participants closed their eyes and stood upright on one leg on the treadmill, while the other leg was allowed to freely flex at the knee and hip (Fig. 1o). They flexed the knee and hip to a target angle, held it for approximately three seconds, returned to the starting position, and then reproduced the target angle for three seconds (Fig. 1o). The target angles were visually determined by a research team member and varied randomly within the following ranges for all participants: 40° to 90° for knee flexion and 10°–45° for hip flexion [17]. The average angle of the middle second was defined as the target/reproduce angle. All participants underwent three trials of knee and hip flexion, which were recorded using an eight-camera Oqus system at 200 Hz (Qualisys, Gothenburg, Sweden).

#### Kinematics, Kinetics and Landing Strategies Collection

A five-minute treadmill familiarization at a self-selected speed was conducted prior to kinematic and kinetic data collection. This process ensured that participants were comfortable with treadmill running, reducing the likelihood of learning effects. All participants reported prior exposure to treadmill running as part of their regular training routines. Participants then ran on a level treadmill at a fixed speed of 10 km/h for 5 min, with kinematic, kinetics and landing strategies collected synchronously for 20 s during the final 2 min of the run to ensure steady-state conditions, using an eight-camera Oqus system at 200 Hz and an instrumented treadmill at 2000 Hz (h/p/cosmos sports & medical GmbH, Nussdorf-Traunstein, Germany). After this pre-test, participants completed a simulated half-marathon at their preferred speed, aiming to complete it as quickly as possible (average time: 2:00:08 ± 0:11:16; males: 1:53:20 ± 0:10:09; females: 2:06:13 ± 0: 08: 32). All participants wore their own spandex tights and running shoes for the tests. Identical kinematic and kinetic measurements were conducted immediately post, one day post, and two days post-run.

### Data Analyses

Torque data was transferred from the Isomed 2000 to a computer and analyzed. The peak torque of the middle three repetitions were used for knee flexors and extensors for statistical analysis. Peak ground reaction forces, impulse, contact time, joint angles and moments during the stance phase of running, were calculated using a lower extremity model with the commercial software Visual3D (C-Motion, Germantown, MD, USA). Kinematics and joint moments were low pass filtered at 10 Hz. Ground reaction forces were low pass filtered at 30 Hz, which selected based on prior studies [18]. The stance phase of running was identified based on the vertical ground reaction force data (20 N threshold), from initial ground contact to foot-off. The average peak value of ten consecutive gait cycles was used for analysis. The knee and hip joint position sense were calculated based on the differences between the target and replicated angles, quantified using absolute (AE) and relative (RE) angular errors. AE is the absolute difference between the target angle and the replicated angle. RE represents the signed arithmetic difference, accounting for accuracy in terms of the direction of the error.

### Statistics Analyses

Statistical analysis was conducted using SPSS software (IBM Corp., Armonk, NY, USA) and Prism v.9.0 (GraphPad Software, San Diego, CA, USA). The normality of the data was assessed using the Shapiro-Wilk test. Gender by time interactions were analyzed using repeated-measures ANOVA with a Bonferroni correction for multiple comparisons. Data are presented as mean ± SEM (Standard error of the mean). The significance level was set at *p* < 0.05.

## Results

### Half-Marathon Running Induces Alterations to Neuromuscular Function

To investigate the acute changes in neuromuscular function following a simulated half-marathon (Fig. 1a) in recreational runners, we measured the concentric and eccentric torques of the knee flexors and extensors at a velocity of 60°/s using the Isomed 2000 (Fig. 1b), both pre and immediately post half-marathon. Compared to pre-half-marathon measurements, we observed significant reductions in both concentric (Flexors: Pre: 1.49 ± 0.50Nm/kg, Post: 1.34 ± 0.54Nm/kg, *p* < 0.001; Extensors: Pre: 2.19 ± 0.73Nm/kg, Post: 2.00 ± 0.82Nm/kg, *p* < 0.001) and eccentric (Flexors: Pre: 1.67 ± 0.68Nm/kg, Post: 1.34 ± 0.62Nm/kg, *p* < 0.001; Extensors: Pre: 2.44 ± 0.13Nm/kg, Post: 1.96 ± 0.12Nm/kg, *p* < 0.001) peak torques of the knee flexors and extensors post running (Fig. 1c). Secondly, we examined the functional torque ratios (eccentric knee flexor torque to concentric knee extensor torque) and the conventional torque ratios (concentric knee flexor torque to concentric knee extensor torque). Compared to pre-half-marathon measurements, significant reductions in functional torque ratios (Pre: 0.78 ± 0.27, Post: 0.68 ± 0.22, *p* < 0.001) were observed post running (Fig. 1d). However, the conventional torque ratios did not change post-half-marathon relative to pre-half-marathon measurements (Fig. 1d).

Additionally, we used an eight-camera Oqus system to measure the active reproduction of knee and hip joint position sense (Fig. 1e). We observed an increase in both the absolute angular errors (the absolute difference between the target angle and the replicated angle) of the knee (Pre: 1.6 ± 0.1°, Post: 3.0 ± 0.2°, *p* < 0.001) and hip (Pre: 1.5 ± 0.2°, Post: 2.6 ± 0.2°, *p* < 0.001) and the relative angular error (the signed arithmetic difference) of the hip (Pre: 0.82 ± 0.22°, Post: 1.61 ± 0.32°, *p* = 0.026) following the half-marathon (Fig. 1f).

### Half-Marathon Running Impacts Landing Strategies

To investigate the changes in peak vertical ground reaction forces, impulse, and contact time (Fig. 1g) following a simulated half-marathon, we examined the parameters mentioned above during the stance phase of running using the h/p cosmos instrumented treadmill pre and post a half-marathon. We observed an increase in contact time (*p* = 0.006) (Fig. 1h) and a decrease in peak vertical ground reaction forces (*p* < 0.001) (Fig. 1i) and impulse (*p* < 0.001) (Fig. 1j) after the half-marathon.

### Half-Marathon Running Elicits Lower Extremity Joint Mechanics Changes

We investigated the acute changes in lower extremity kinematics and kinetics (Fig. 1kn) separately for males and females following a simulated half-marathon, using an eight-camera Oqus system and an instrumented treadmill. For hip joint moments, we observed increased peak hip extension moments (Female: *p* < 0.001; Male: *p* < 0.001), decreased peak hip abduction moments (Female: *p* < 0.001; Male: *p* = 0.026), and reduced hip external rotation moments (Female: *p* = 0.002; Male: *p* = 0.039) post-half-marathon compared with pre (Fig. 1l). Regarding hip joint angles, increased peak hip adduction was observed in both females (*p* = 0.021) and males (*p* = 0.027), with a gender difference showing increased peak hip internal rotation in males (*p* = 0.011) and no change in females (*p* = 0.468) post-half-marathon compared to pre (Fig. 1m). Furthermore, we detected a higher peak knee abduction angle in females (*p* = 0.017) and lower peak knee abduction moments (Female: *p* < 0.001; Male: *p* = 0.043) in both genders post-half-marathon (Fig. 1o, p).

### Muscle Capacity Recovered Within One Day and Increased Within Two Days

**Fig. 2:**
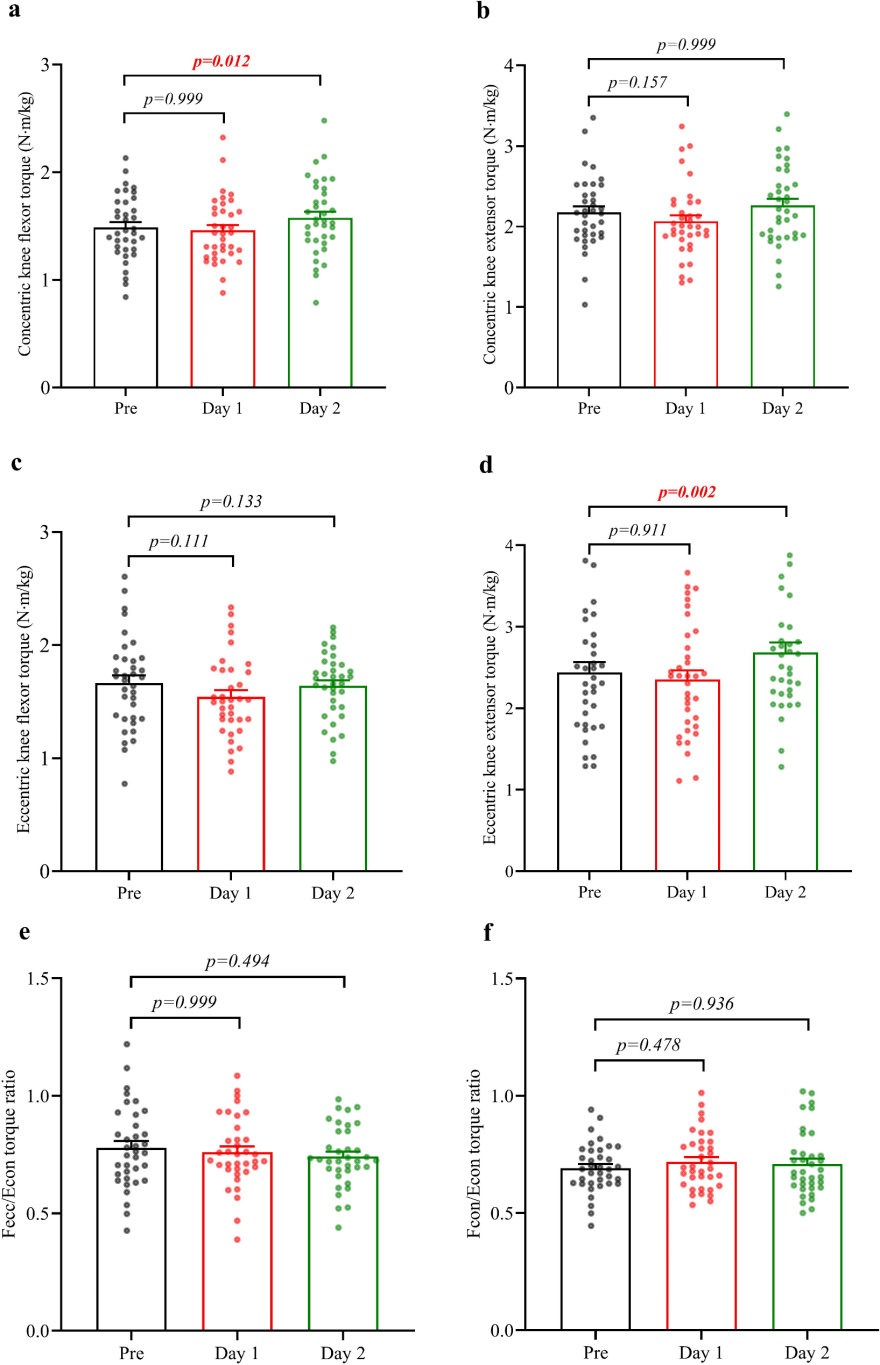
Changes in peak knee flexor and extensor torques (**a**,** b**,** c**,** d)**, functional peak torque ratio **(e)** and the conventional peak torque ratio **(f)** pre, one day post, and two days post half-marathon. The peak torque of the middle three repetitions was used for flexors and extensors for statistical analysis. Data are presented as mean ± SEM (Standard error of the mean). The error bars represent the SEM of the data across participants. Statistical analyses were conducted using two-way repeated measures ANOVA, followed by Bonferroni’s multiple comparison tests. The p-value indicates alterations in torque post-half-marathon compared to pre

We detected no significant differences in the torque of the knee flexors and extensors, nor in the functional torque ratio pre and one day post-half-marathon measurements (Fig. 2). This suggests that within one day post-half-marathon, the torque of the runners’ flexors and extensors had returned to baseline levels. Remarkably, two days post-half-marathon, participants exhibited increased concentric flexors and eccentric extensors torque relative to their baseline measurements (Fig. 2a-d). These data indicate that some indicators showed signs of supercompensation within two days.

### Proprioception Deficit Resolved Within Two Days

**Fig. 3:**
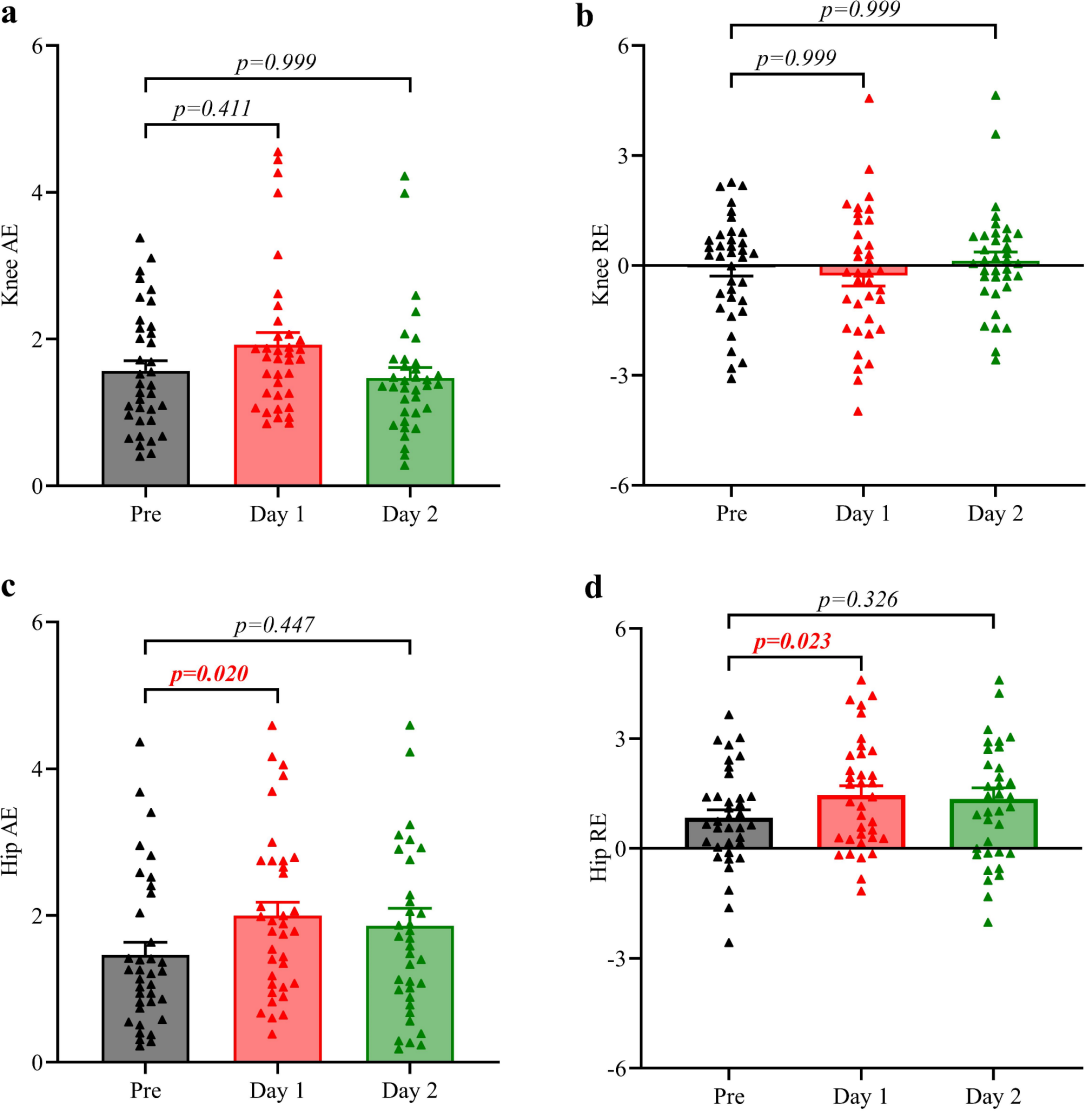
Changes in joint position sense in the knee (**a**,** b**) and hip (**c**,** d**) flexion pre, one day post, and two days post half-marathon. An active reproduction technique was utilized, with both absolute (AE) and relative (RE) angular errors calculated between the target and replicated angles. The mean value of three trials was used for AE and RE calculations. Data are presented as mean ± SEM (Standard error of the mean). The error bars represent the SEM of the data across participants. Statistical analyses were performed using two-way repeated measures ANOVA, followed by Bonferroni’s multiple comparison tests. The p-value indicates alterations in joint position sense post-half-marathon compared to pre

To investigate the recovery time in neuromuscular control following a half-marathon, we assessed knee and hip joint position sense pre, one day, and two days post half-marathon running. We observed no significant differences in knee angular error one day or two days post-half-marathon compared with baseline measurements (Fig. 3a, b). However, an increase in hip angular error (AE: *p* = 0.020; RE: *p* = 0.023) was noted one day post, which returned to baseline by two days post (Fig. 3c, d). This data indicates that the recovery of hip proprioception lags behind that of the knee joint.

### Landing Strategies and Joint Mechanics Restored Within One Day

**Fig. 4:**
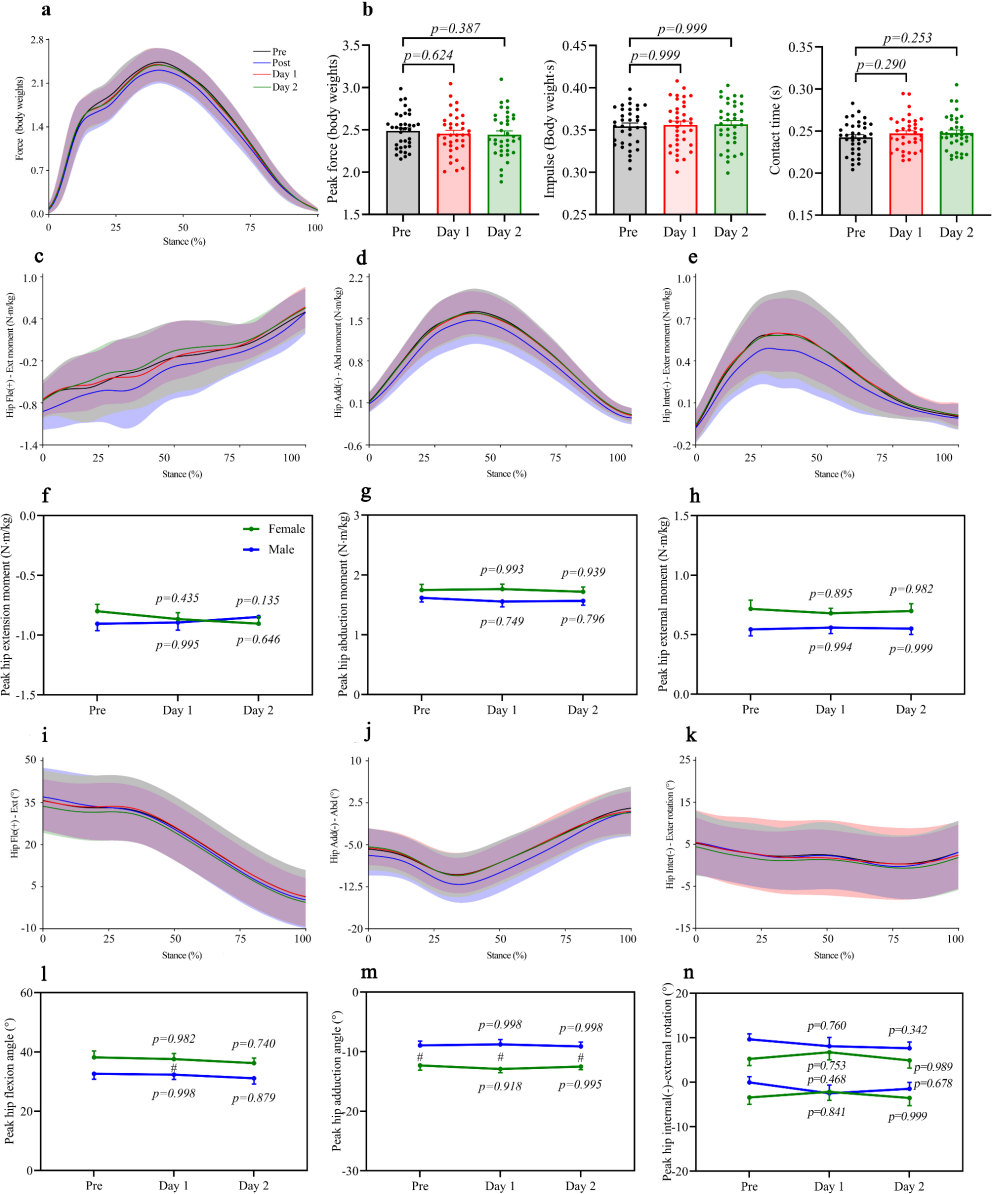
Changes in vertical ground reaction forces, impulse, contact time, hip joint moments, and hip joint angles during the stance phase of running pre and post half-marathon. **a**, Mean values of vertical ground reaction forces from all participants, where the bold line represents the mean and the shaded area represents the standard deviation. **b**, Peak vertical ground reaction forces, impulse, and contact time during the stance phase of running. Mean values of hip joint moments (**c**,** d**,** e**) and hip joint angles (**i**,** j**,** k**) from all participants. Peak hip joint moments (**f**,** g**,** h**) and peak hip joint angles (**l**,** m**,** n**) during the stance phase of running. The peak average value of ten consecutive gait cycles was used for analysis. Data are presented as mean ± SEM (Standard error of the mean). The error bars represent the SEM of the data across participants. Statistical analyses were performed using two-way repeated measures ANOVA, followed by Bonferroni’s multiple comparison tests. The p-value indicates alterations in landing strategies and joint mechanics post-half-marathon compared to pre. # indicates significant gender differences

**Fig. 5:**
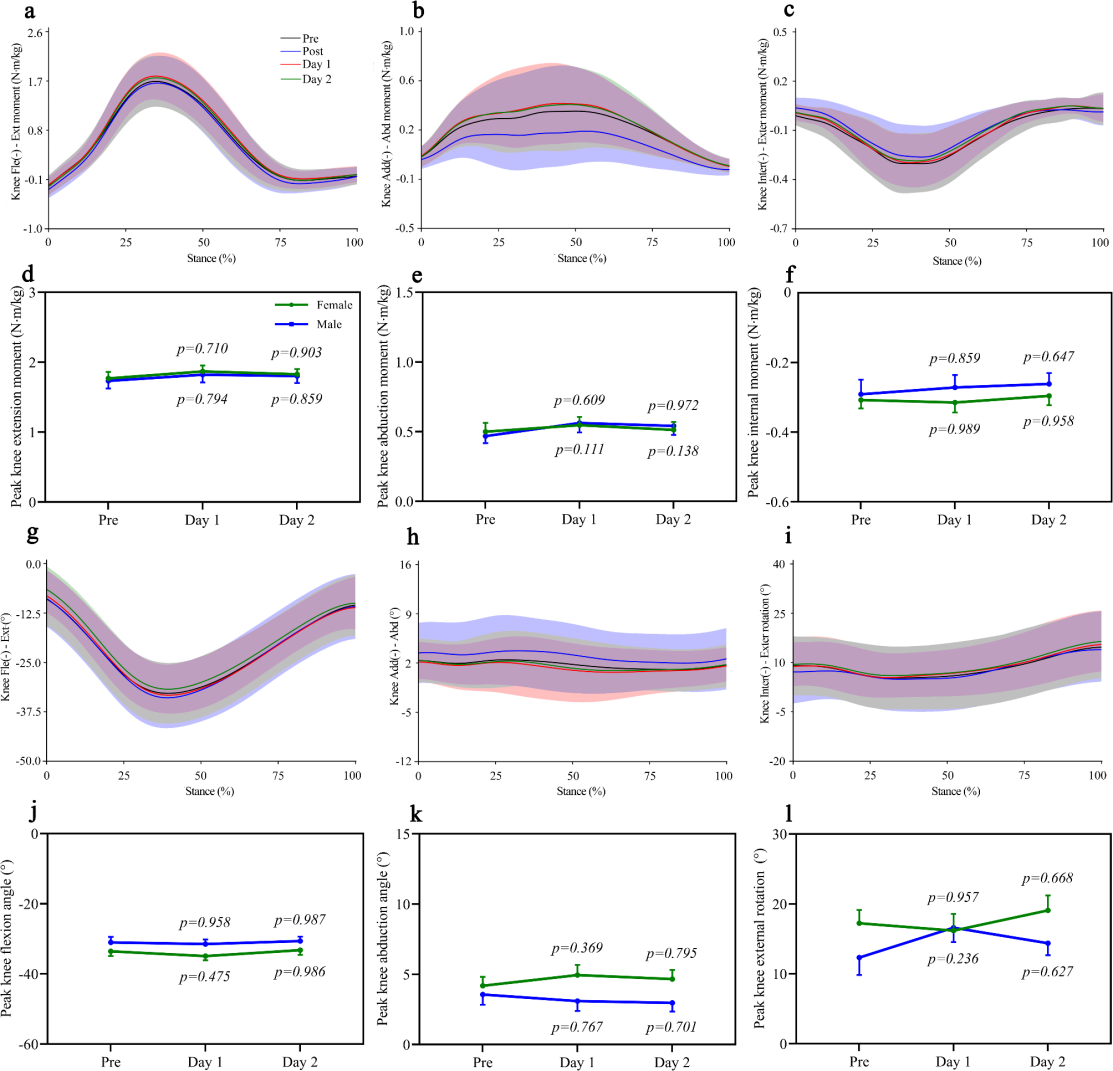
Changes in knee joint moments and knee joint angles during the stance phase of running pre and post half-marathon. Mean values of knee joint moments (**a**,** b**,** c**) and knee joint angles (**g**,** h**,** i**) from all participants. Peak knee joint moments (**d**,** e**,** f**) and peak knee joint angles (**j**,** k**,** l**) during the stance phase of running. The peak average value of ten consecutive gait cycles was used for analysis. Data are presented as mean ± SEM (Standard error of the mean). The error bars represent the SEM of the data across participants. Statistical analyses were performed using two-way repeated measures ANOVA, followed by Bonferroni’s multiple comparison tests. The p-value indicates alterations in joint mechanics post-half-marathon compared to pre

To investigate the time course of recovery for related parameters, we assessed peak vertical ground reaction forces, impulse, and contact time during the stance phase of running at pre, one day post, and two days post-half-marathon (Fig. 4a). No differences were detected in all parameters above, whether one day or two days post-half-marathon compared to pre (Fig. 4b). Similarly, we investigated the recovery time for hip joint (Fig. 4c, d, e, i, j, k) and knee joint (Fig. 5a, b, c, g, h, i) angles and moments following a half-marathon. We found that hip joint (Fig. 4f, g, h, l, m, n) and knee joint (Fig. 5d, e, f, j, k, l) angle/moment parameters returned to baseline within one day, with no further changes observed two days post-half-marathon.

## Discussion

Our observations indicate that running a simulated half-marathon is associated with significant changes in biomechanical parameters. Specifically, we observed reductions in muscular torque and functional torque ratios, alterations in lower extremity joint angles and moments, decreases in ground reaction force and impulse, increased contact time, and deficits in joint position sense. Within one day post-run, most parameters showed recovery to baseline levels, except for hip proprioception, which demonstrated continued impairment. By two days post-run, these changes were fully resolved, with some torque indicators showing signs of supercompensation.

Half-marathon running induces the alterations to neuromuscular function, which probably result from a complex interaction between central and peripheral mechanisms. Prolonged exercise leads to impaired neuromuscular function due to impairments in skeletal muscle fibers’s Ca2^+^ release or sensitivity [19], failure of excitation-contraction coupling [20], as well as a reduction in the capacity of the central nervous system activate muscles [21]. Moreover, we observed impacts landing strategies following a simulated half-marathon running. High external forces are known to cause pain and injury in runners [22]. Therefore, the decrease in ground reaction force maybe an injury prevention strategy. Impulse refers to the product of force and the time over which that force is applied. Increasing the contact time in order to compensate for decreasing ground reaction force may be a compensatory strategy to maintain running impulse [23]. In our study, we detected a decrease in impulse after half-marathon, which means that this compensatory strategy is not sufficient to counteract the effects of fatigue. However, it remains challenging to determine whether these changes in running biomechanics represent a strategy to prevent injuries, a consequence of fatigue-induced performance decline, or a combination of both factors.

Our results suggest that half-marathon running results in alterations in lower extremity joint angle and moment parameters. These alterations can contribute to changes in running gait, potentially influencing injury risk and performance [8, 11]. Joint stiffness can be calculated from the ratio of maximal joint moment to maximum joint angle using the spring-mass model [24]. In the sagittal plane of the hip joint, we observed an increased extension moment and no significant change in the peak hip flexion angle post-half-marathon, indicative of increased hip joint stiffness in this plane. Conversely, we noted a decrease in peak hip moment and an increase or no change in peak hip angular displacement in the coronal and transverse planes, suggesting reduced tissue stiffness in these planes. Running primarily involves hip movement in the sagittal plane, with hip flexion during the swing phase as the leg moves forward and hip extension during the stance phase to propel the body forward. Therefore, we speculate that the variation in stiffness across different planes suggests that fatigue levels in the hip joint vary, being higher in the sagittal plane. Knee joint moment parameters were less affected by the half-marathon; only a lower peak knee abduction moment was observed immediately post-half-marathon. Furthermore, only the female knee valgus angle showed a significant increase in joint angle parameters. Increased knee valgus is associated with the risk of anterior cruciate ligament injuries [25]. Research indicates that females are at a higher risk of lower extremity injuries compared to their male counterparts, particularly suffer anterior cruciate ligament injury at a four to six fold higher rate [25–27]. Our findings suggest that females exhibit greater knee valgus during the stance phase of running post-half-marathon, potentially leading to decreased joint stability and an increased risk of injury.

Our above-mentioned findings identify the acute changes in lower extremity biomechanics following a simulated half-marathon, which is partly consistent with previous studies on biomechanical risk factors for patellofemoral pain syndrome. Biomechanical risk factors for this syndrome in distance runners include abnormalities in lower extremity biomechanics [4]. Runners who develop patellofemoral pain syndrome exhibit significantly greater peak hip adduction angles [28–30], increased peak hip internal rotation angles [31, 32], reduced peak hip abduction moments [33], diminished peak knee flexion [34], increased peak knee external rotation [35], greater peak knee adduction angles [36], and longer stance times [22] during the stance phase of running. Additionally, individuals with persistent patellofemoral pain syndrome demonstrate reduced knee extension peak torque [22, 37] and impaired proprioceptive function. Patellofemoral pain syndrome has the highest prevalence among running-related musculoskeletal injuries in runners [2]. We speculate that reductions in knee extension peak torque, impaired proprioceptive function, increases in peak hip adduction, decreases in peak hip abduction moments, and prolonged stance times during the stance phase of running-induced after a half-marathon may increase the risk of patellofemoral pain syndrome injury.

Training for success involves striking a balance between reaching peak performance and avoiding the pitfalls of overtraining [38, 39]. All biomechanics-related indicators we monitored recovered within two days, therefore, we recommend that runners avoid engaging in higher-intensity physical activities within two days after completing the half marathon, as during this time window, runners are at a greater risk of injury. It is not possible to establish a full recovery until all parameters return to the baseline. Therefore, the final recovery indicator is worthy of attention. In our study, we found that the recovery of hip joint parameters behind that of the knee joint. The primary muscles responsible for accelerating the body’s center of gravity during the ground phase of running are the hip joint extensors, with the knee joint extensors also playing a significant, though lesser role [40]. Therefore, our observed results may indicate that the hip joint exhibits greater fatigue after half-marathon running. Runners can add some hip-dominant functional training to their daily lower-limb strength training to improve the fatigue resistance of the hip joints during running.

Previous studies have indicated that if an athlete has not recovered within three days, they are likely overstrained and in an overreached state [38]. This suggests that, from a biomechanical perspective, participating in a half-marathon may not result in overtraining. Another point worthy of attention is that within two days some torque indicators have shown signs of super-compensation. This result means neuromuscular performance has been shown to be potentiated within two days following half-marathon running. Previous studies have demonstrated that various measures of neuromuscular performance can be improved for up to two days after resistance exercise [41]. However, there is currently few research on the delayed neuromuscular potentiation effects after endurance training. Some research suggests that increased muscle torque can improve general sports skills, contribute to overall athletic performance, and reduce injury risk [41, 42]. Therefore, further investigation examining the effects of long-distance running on neuromuscular performance would be particularly beneficial for practitioners to evaluate the effectiveness of this strategy in marathon sports.

This study had the limitation that employed a treadmill-based simulated half-marathon protocol. While this setup ensures controlled conditions, it does not fully replicate a real half-marathon race on the road outdoor. Secondly, this study is the use of a fixed running speed (10 km/h) for kinematic and kinetic data collection. It likely resulted in differing relative exercise intensities between participants, but this approach ensured that data were collected at the same running speed. Lastly, participants ran a half-marathon on a laboratory-instrumented treadmill at their preferred speed, aiming to complete it as quickly as possible. While this design may reflects real conditions, the absence of physiological measures such as heart rate or oxygen consumption data limits the ability to fully account for intensity differences. Future studies could incorporate additional metrics to better quantify and standardize effort levels.

## Conclusion

Overall, our study identified changes in neuromuscular function, landing strategies, and lower extremity joint mechanics following a simulated treadmill half-marathon, which may increase the risk of injury. However, these effects are temporary and disappear within two days after running. By the second day, we also observed super-compensation in thigh muscle torques. Conducting research on the acute changes in lower extremity biomechanics and the recovery time following a simulated half-marathon is essential to optimize training responses and prevent overtraining for runners.

## Data Availability

Requests for additional data and material should be addressed to Wenjin Wang at wangwenjin0213@163.com.
